# Fluoride Increases Superoxide Production and Impairs the Respiratory Chain in ROS 17/2.8 Osteoblastic Cells

**DOI:** 10.1371/journal.pone.0100768

**Published:** 2014-06-25

**Authors:** Brenda Lorena Fina, Mercedes Lombarte, Juan Pablo Rigalli, Alfredo Rigalli

**Affiliations:** 1 Bone Biology Laboratory, School of Medicine, Rosario National University, Rosario, Santa Fe, Argentina; 2 Institute of Experimental Physiology, Consejo Nacional de Investigaciones Científicas y Técnicas (CONICET), Rosario, Santa Fe, Argentina; University of Newcastle, United Kingdom

## Abstract

It is known that fluoride produces oxidative stress. Inflammation in bone tissue and an impairment of the respiratory chain of liver have been described in treatments with fluoride. Whether the impairment of the respiratory chain and oxidative stress are related is not known. The aim of this work was to study the effects of fluoride on the production of superoxide radical, the function of the respiratory chain and the increase in oxidative stress in ROS 17/2.8 osteoblastic cells. We measured the effect of fluoride (100 µM) on superoxide production, oxygen consumption, lipid peroxidation and antioxidant enzymes activities of cultured cells following the treatment with fluoride. Fluoride decreased oxygen consumption and increased superoxide production immediately after its addition. Furthermore, chronic treatment with fluoride increased oxidative stress status in osteoblastic cells. These results indicate that fluoride could damage bone tissue by inhibiting the respiratory chain, increasing the production of superoxide radicals and thus of the others reactive oxygen species.

## Introduction

Fluoride (F-) is a typical double-edged weapon for human beings. On one hand, its daily administration prevents from tooth cavities and has a mitogenic action on osteoblasts [1]. On the other hand, F- chronic exposure has been demonstrated to be toxic and to cause fluorosis. Several studies have described an increase in bone mineral density in treatments with F- [2]. However, the bone is poorly mineralized and exhibits inflammatory foci [3], which could explain the lack of beneficial effects in treatments with sodium fluoride (NaF). It has been demonstrated that 100 µM of F- decreased the proliferation of osteoblasts and induced apoptosis through the production of reactive oxygen species (ROS) [4].

The generation of ROS, lipid peroxidation and altered antioxidant defence systems are considered to play an important role in the toxic effects of F-. Although damaging effects of F- and ROS production are well documented, the cellular mechanisms by which F- induces ROS formation in bone tissue is still unknown [5].

Mitochondria are considered to be the major source of intracellular reactive oxygen species [6]. The mitochondrial electron transport chain is a major site of superoxide radicals’ production, followed by formation of hydrogen peroxide (H_2_O_2_), which can be converted into the reactive hydroxyl free radical causing oxidative damage [7]. Most oxygen consumed (98%) by cells is used in mitochondria [8] so a key parameter of mitochondrial function is the value of oxygen uptake rate (VO_2_). Changes in the oxygen availability or alterations in the electron transport can increase superoxide production.

Previous studies performed in bacteria have demonstrated that F- can be extracelularly protonated to form hydrofluoric acid that freely diffuses through the membrane [9]. Therefore, F- could enter mitochondria following a similar mechanism. We have previously demonstrated that the treatment with F- produced oxidative stress and decreased VO_2_ in liver [10]. However the link between the two processes was not found and there is scarce evidence about the effects of therapeutically used concentrations of F- on bone tissue or cells. The aim of this study was to assess the effects of F- on the production of superoxide radical, oxygen consumption and oxidative stress in ROS 17/2.8 osteoblastic cells. Fluoride concentrations used in the experiments described in this paper were within the range of plasma concentrations (10–100 µM) found after the intake of a therapeutic dose of F- (3–20 mgF-/Kg bw. day) [11], [12] or water with high fluoride concentration [13].

## Materials and Methods

### Cell Culture

ROS 17/2.8 osteoblastic cell line was developed by Dr. Gideon Rodan and kindly donated by Dr. Ricardo Boland (Universidad Nacional Del Sur, Argentina) [14], [15]. Cells were grown in DMEM/Ham’s F-12 medium (1∶1) (Invitrogen, Carslbad, CA, USA) containing 10% inactivated fetal bovine serum (PAA, Pasching, Austria), 2 mM Glutamine (Invitrogen), 100 Units Penicillin/mL and 100 µg streptomycin/mL (Invitrogen), at 37°C in a humidified atmosphere of 5% CO_2_.

### Fluoride Treatments

#### Acute experiments

These experiments were carried out to study the effects of F- when it immediately contacts with cells and mitochondria. For this purpose, F- was added to cells or isolated mitochondria while were respiring. Briefly, subconfluent ROS 17/2.8 cells were trypsinized and immediately transferred to an oxygen chamber at a density of 1.6×10^6^ cells/mL. Basal VO_2_ was measured for 1 min. Afterwards, the effect of F- (10, 50 or 100 µM) on VO_2_ was measured. The experiment was repeated seven times for each F- concentration. Then, isolated mitochondria from subconfluent ROS 17/2.8 cells were obtained and resting mitochondrial VO_2_ (state 4) and active VO_2_ (state 3) were measured before and after the addition of 100 µM of F-. Finally, superoxide production was studied in isolated mitochondria before and after the addition of 100 µM of F-.

#### Chronic experiment

This treatment mimics a situation where daily doses of F- are chronically consumed. It has been reported that after an oral F- dose there is a peak plasma concentration of approximately 100 µM followed by a fast return to basal concentration levels [11], [16]. In order to reproduce more precisely the *in vivo* situation, ROS 17/2.8 cells were exposed to a daily 15-min pulse of NaF (100 µM, F-treated cells) or water (control cells) for three consecutive days. After each pulse, culture medium was removed and cells were further incubated for 24 h in fresh medium. After three days, cells were released by trypsinization and used for VO_2_ measurements in intact cells, activity of respiratory chain complexes and oxidative stress indexes. The volume of F-solution (treated cells) or distilled water (control cells) was 0.1% of the total culture medium. The chronic experiment was repeated three times.

Oxidative stress indexes were measured in Control and F- groups. Isolated mitochondria were obtained from control and F-treated groups (see below) to measure respiratory complexes activities.

The results of the chronic experiment are expressed as percentage of the respective control group (water-exposed), considered as 100%.

### Measurement of Oxygen Uptake Rate (VO_2_)

VO_2_ was measured in cell suspensions and in isolated mitochondria using a hermetically sealed oxygen measure chamber equipped with a Clark-type electrode maintained at 37°C [17]. Data were recorded with software designed in the laboratory (Biomedical data acquisition suite 1.0). VO_2_ from cell suspensions was measured in growth medium. VO_2_ from isolated mitochondria was measured in a reaction medium consisting of 0.23 M mannitol, 0.07 M sucrose, 20 mM Tris-HCl, 1 mM EDTA, 3 mM MgCl_2_ and 5 mM KH_2_PO_4_/K_2_HPO_4_ (pH 7.40). Succinate 6 mM was used as substrate to measure resting respiration (state 4) and 1 mM ADP was added to measure active respiration (state 3). Respiratory control was calculated as the relationship between state 3 respiration and state 4 respiration. To be sure mitochondrial respiration was being measured; 25 mM of KCN (complex IV inhibitor) was added at the end of the measurement.

### Isolation of Mitochondria

Mitochondria were isolated from ROS 17/2.8 cells as previously described by Boveris A. [18]. Briefly, cells were lysed by sonication in a MSTE medium consisting of 0.23 M mannitol, 0.07 M sucrose, 10 mM Tris-HCl, 1 mM EDTA, pH 7.40 (3 pulses, 5 seconds, 30% amplitude) using a Vibra-Cell VCX130 device (Sonics & Materials, Newton, CT, USA). Resulting lysates were centrifuged at 500 g for 10 min to discard nuclei and cell debris. The sediment was discarded and the supernatant was centrifuged at 11000 g for 10 min to obtain the enriched mitochondrial fraction. Purity of isolated mitochondria was assessed by determining lactate dehydrogenase activity; only mitochondria with less than 10% impurity were used [19], [20]. The total protein concentration of the obtained fractions was measured using a commercial kit based on the red pyrogallol-molibdate method [21] (ProtiU/LCR, Wiener Lab, Rosario, Argentina).

### Preparation of Submitochondrial Particles

Submitochondrial particles were obtained for measurement of the activity of respiratory complexes and superoxide production. For this purpose, previously obtained mitochondria were resuspended in MSTE buffer, frozen and thawed three times, and homogenized through the passage of the suspension through a 27G needle 15 mm in length and 0.1 mm in outer diameter [19], [20].

### Activity of Respiratory Complexes

The determination of the activities of NADH-cytochrome c reductase (Complex I–III) and succinate-cytochrome c reductase (Complex II–III) was based on the reduction of cytochrome c^3+^ to cytochrome c^2+^ and was followed spectrophotometrically at 550 nm for 2 min at 30°C. The reaction mixture was composed of 100 mM buffer H_2_KPO_4_/HK_2_PO_4 _pH 7.40, mitochondrial membranes (0.02 mg protein/ml), 0.5 mM KCN (to inhibit the activity of complex IV), 25 µM cytochrome c^3+^, and 5 mM NADH or 0.2 mM succinate. The activities were calculated as µmol.min^−1^.mg protein^−1^ [22].

The determination of the activity of cytochrome oxidase (Complex IV) was based on the oxidation of cytochrome c^2+^ to cytochrome c^3+^ and followed spectrophotometrically at 550 nm for 1 min at 30°C. The reaction mixture was composed of 100 mM buffer KH_2_PO_4_/K_2_HPO_4 _pH 7.40, mitochondrial membranes (0.1 mg protein/ml) and 40 µM cytochrome c^2+^. As this reaction follows a pseudo-first order kinetic mechanism, the constant (k = min^−1^) of the one-phase exponential decay was used as a measure of the activity of the complex, expressed as min^−1^.mg protein^−1^ [23].

### Measurement of Superoxide Production by Submitochondrial Particles

Superoxide production was measured by the SOD-inhibitable oxidation of adrenaline to adrenochrome [24]. The assay medium (pH 7.40) contained 0.23 M mannitol, 0.07 M sucrose, 20 mM Tris-HCl, 1 mM adrenaline and 0.5 mg/mL submitochondrial particles. Superoxide production was iniciated with 7 mM succinate. Formation of adrenochrome was followed spectrophotometrically at 480 nm (ε = 4.0 mM/cm) for 2 min.

For the acute experiment, superoxide production was measured before and after the addition of 100 µM of F-.

### Measurement of Glutathione Peroxidase (GPx) and Catalase (CAT) Activities

Glutathione peroxidase (GPx) activity was determined in cell lysates following the method of Lawrence and Burk [25] employing 0.25 mM hydrogen peroxide as substrate. The assay medium contained 50 mM buffer KH_2_PO_4_/K_2_HPO_4 _pH 7.40, 1 mM EDTA, 1 mM NaN_3_, 0.2 mM NADPH, 1 mM GSH, and 1 U/ml glutathione reductase. The consumption of NADPH was followed spectrophotometrically at 340 nm. The resulting activity was expressed in µmol.min-1.mg of protein-1.

Catalase (CAT) activity was evaluated following Aebi’s method [26]. The principle of the assay is based on the determination of the rate constant of hydrogen peroxide decomposition by catalase enzyme. The decomposition of the substrate hydrogen peroxide was monitored spectrophotometrically at 240 nm for 3 min in a reaction medium consisting of 100 mM phosphate buffer (pH 7.40) and 20 mM hydrogen peroxide.

### Measurement of Malondialdehyde (MDA)

MDA, the marker of extent lipid peroxidation, was estimated as thiobarbituric acid reactive substances (TBARS) level by the method of Ohkawa [27]. The principle of the method is based on the spectrophotometric measurement of the complex that appeared during thiobarbituric acid’s reaction with MDA. Briefly, cells were lysed by sonication (3 pulses, 5 seconds, 30% amplitude). Resulting lysates were incubated for 1 h at 95°C in a reaction medium consisting of 14 mM sodium dodecyl sulfate, 1.25 M acetic acid and 18 mM thiobarbituric acid. Then samples were centrifuged at 1000 g for 5 min and supernatants were subjected to deproteinization with 0.31 M of trichloroacetic acid. Finally, samples were centrifuged at 1000 g for 10 min and the absorbances of supernatants were measured at 532 nm. The amount of TBARS was expressed in nmol.mg of protein-1.

### Measurement of Oxidized/Total Glutathione Ratio

Oxidized/total glutathione ratio (GSSG/TGSH) was calculated as the quotient between the intracellular oxidized (GSSG) and total glutathione (TGSH) contents determined spectrophotometrically by the enzymatic recycling procedure of Tietze [28], as modified by Griffith [29]. Briefly, cells were scraped in phosphate saline buffer (125 mM, pH 7.50) supplemented with EDTA (6.3 mM) and lysed by sonication (3 pulses, 5 seconds, 30% amplitude). Resulting lysates were subjected to deproteinization with sulfosalicylic acid (10%) and used for the determination of TGSH or derivatized by incubation with 2-vinylpyridine and triethanolamine (3 µl and 5 µl for 100 µl of deproteinized supernatant respectively) for 1 h at 30°C. Derivatized samples were used for GSSG. Reactions were carried out in phosphate saline buffer-EDTA as above described. NADPH (0.21 M) and dithionitrobenzoic acid (0.6 mM) were used as substrates. 2 and 20 µl of sample were used for TGSH and GSSG respectively and reactions were started by addition of glutathione reductase (0.5 units). TGSH and GSSG contents were quantified through the rate of formation of a coloured derivative of dithionitrobenzoic acid measured spectrophotometrically at 412 nm.

### Statistical Analysis

Differences among groups were analysed with One-way analysis of variance (ANOVA) followed by Bonferroni’s post test. Students t-test was used to compare two independent samples. Differences were considered significant if p<0.05. All data in text and figures are provided as mean ± SD. Shapiro and Wilk test demonstrated normal distribution of the samples and Fligner test demonstrated equality of variances of each group. Therefore, parametric tests were used. Data analyses were performed with the package agricolae [30] for R.2.14.1 [31].

## Results

### Acute Experiments

**Figure 1:**
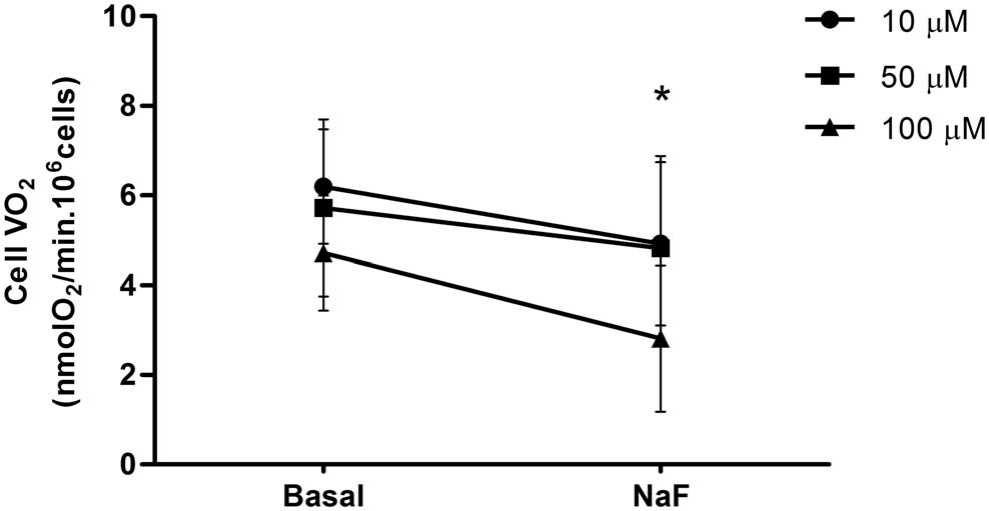
Oxygen uptake rate (VO_2_) in ROS 17/2.8 cells. VO_2_ in the absence (Basal) or presence (NaF) of different concentrations of F- (10, 50, 100 µM). Points and segments represent mean and SD respectively. *Significant differences compared to Basal VO_2_ (before the exposure to F), Paired Student’s t-test, n = 7, p<0.05.

After the addition of 10, 50 or 100 µM of F- a significant decrease in cells VO_2_ was observed for all F- concentrations (Figure 1). The most important inhibition (40.4%) was assessed when cells were exposed to 100 µM of F-. On the basis of the results obtained, we decided to use the highest F- concentration in subsequent experiments.

**Figure 2:**
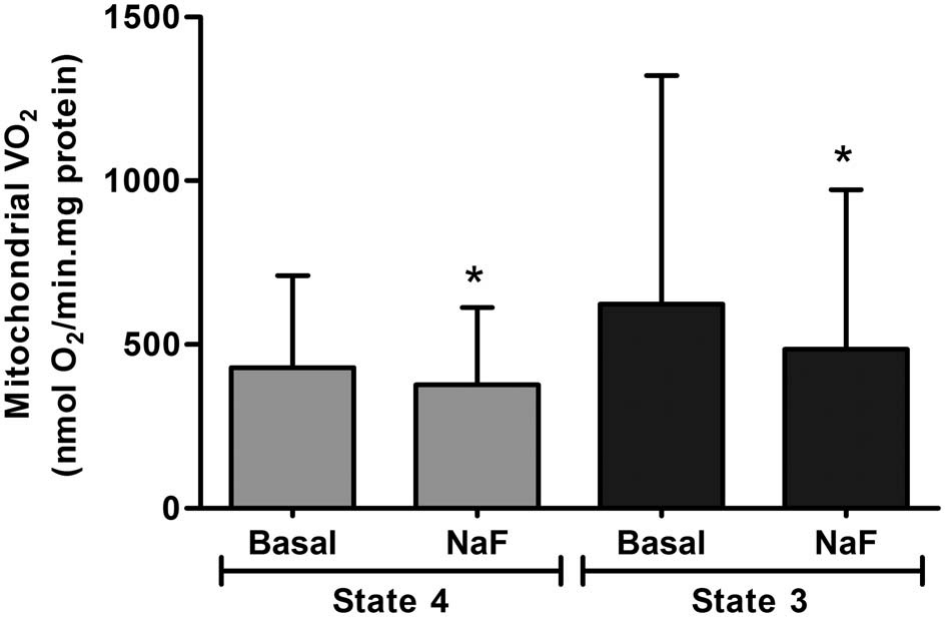
Oxygen uptake rate (VO_2_) in isolated mitochondria. Fluoride effects on VO_2_ of isolated mitochondria in states 4 and 3 in the absence (Basal) or presence of 100 µM of F- (NaF). Bars and segments represent mean and SD respectively. *Significantly different from Basal of the corresponding state. Paired Student’s t test, n = 12, p<0.05.

The addition of 100 µM of F- to active mitochondria produced a significant decrease in mitochondrial respiration, both in state 4 and state 3 (Figure 2). These results coincided with previous VO_2_ inhibition observed in the osteoblasts and demonstrated that F- inhibits the respiratory chain immediately after its addition.

**Figure 3:**
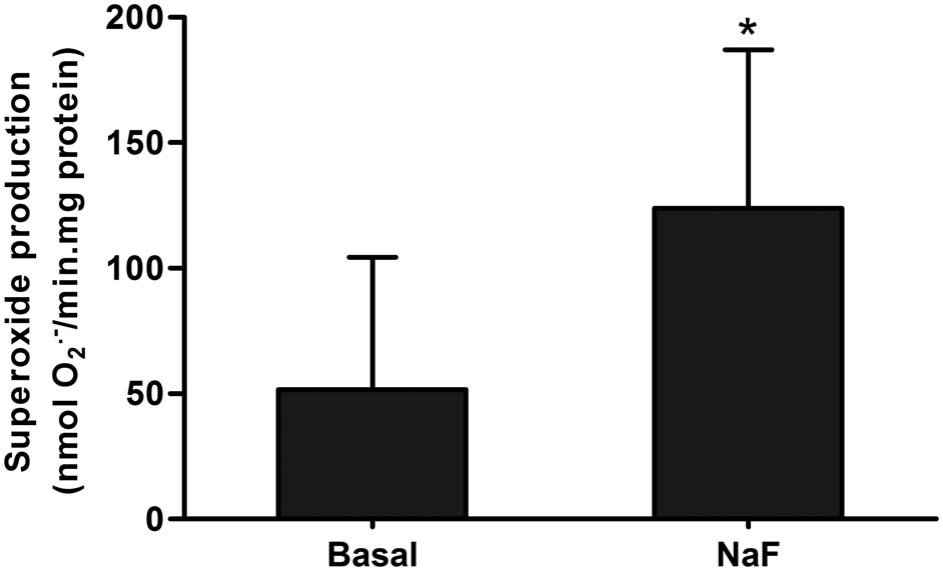
Superoxide production by isolated mitochondria. Fluoride effects on superoxide production of submitochondrial particles before (Basal) and after adding 100 µM of F- (NaF). Bars and segments represent mean and SD respectively. *Significantly different of NaF compared to Basal. Paired Student’s t test, n = 8, p<0.05.

Finally, superoxide production in the absence or presence of F- was assessed to verify whether fluoride-inhibition of the respiratory chain increases the production of superoxide radicals. The results shown in Figure 3 demonstrate a significant increase in superoxide production after the addition of 100 µM of F- to submitochondrial particles.

### Chronic Experiment

**Figure 4:**
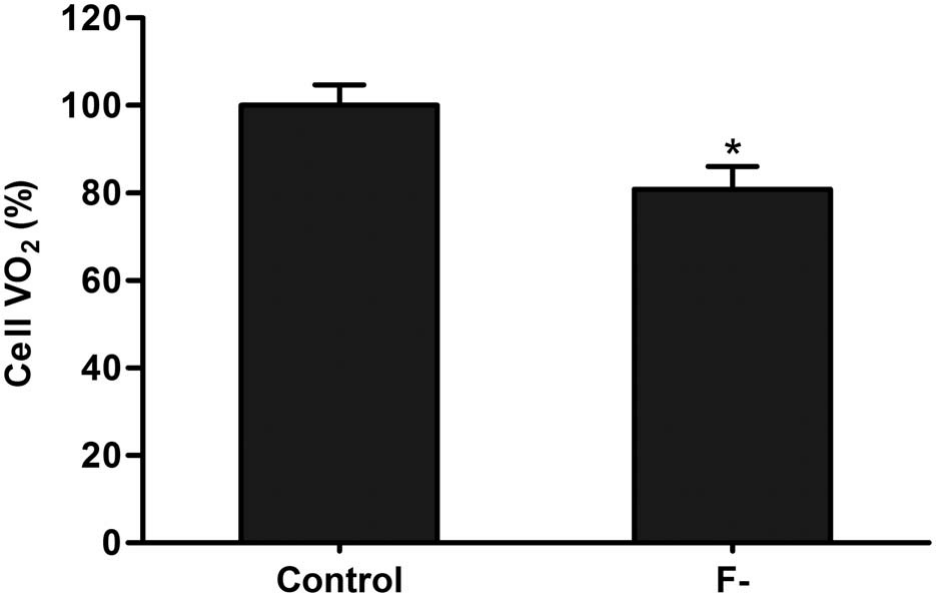
Oxygen uptake rate (VO_2_) after chronic exposure to fluoride. VO_2_ in ROS 17/2.8 cells after chronic treatment with15-min daily pulses of NaF 100 µM (F-) or distilled water (Control) for three consecutive days. Measurements (n = 3) were performed in duplicate and data are presented as percentage (%) of the Control group. Bars and segments represent mean and SD respectively. *Significantly different to Control group, Student’s t test, p<0.05.

F- decreased VO_2_ of osteoblastic cells after 3 pulses of NaF 100 µM of 15 min duration (Figure 4).

The analysis of mitochondrial complexes revealed that there was a decrease of all respiratory complexes activities after the third pulse of F- in F- group. A significant inhibition along time was observed for complex IV reaching an 80% inhibition in F- group (Table 1).

**Table 1:** Respiratory complexes activities of isolated mitochondria.

	Control	F-
**C I–III**	100±28.6	72±28.0
**C II–III**	100±11.7	66±31.6
**C IV**	100±13.3	20±26.2*

Activities of complex I–III (C I–III), complex II-III (C II–III) and complex IV (C IV) of isolated mitochondria from experimental groups expressed as percentage of the Control group. Data are shown as mean ± SD (n = 3). *Significantly different to Control, Student’s t test, p<0.05.

Finally, oxidative stress measurements revealed a significant increase in the levels of TBARS of F-exposed cells compared to Control group (Table 2). The GSSG/TGSH ratio was also increased in cells exposed to F-. Finally, no changes in antioxidants enzymes activities were observed after three pulses with F-.

**Table 2:** Oxidative stress indexes of ROS 17/2.8 cells.

	Control	F-
**CAT**	100±35.1	97±30.2
**GPx**	100±62.7	118±63.0
**TBARS**	100±13.4	181±61.8*
**GSSG/TGSH**	100±3.1	140±7.7*

Catalase (CAT) and glutathione peroxidase (GPx) activities, TBARS levels and GSSG/TGSH ratio in Control and F-treated ROS 17/2.8 cells. Data are expressed as percentage (%) of the Control group and represent three different biological experiments (n = 3). Values are mean ± SD. *Significantly different to Control group, Student’s t test, p<0.05.

## Discussion

The increase in oxidative stress damage caused by F- is well documented [32], but the mechanisms involved in ROS generation are still unknown. One possible explanation is that F- could trigger oxidative stress via inhibition of the pentose phosphate oxidative pathway [33]. In addition, F- induced apoptosis by oxidative stress-induced lipid peroxidation, causing the release of cytochrome c through HL-60 cells mitochondria [34]. Presently, no mechanism for mitochondrial ROS generation by F- in osteoblasts has been proposed. The present contribution has been aimed at investigating whether F- could modify the activity of the respiratory chain in osteoblasts-like cells, changing the rate of production of oxygen reactive species.

We observed that as soon as F- reaches the cells (acute experiment), it significantly inhibits their respiration measured as VO_2_. Also, F- inhibits mitochondrial VO_2_ in both state 4 and state 3 and increases superoxide production. According with these results, the oxygen uptake rate also decreased in chronic treatments with F-. The increase in superoxide radicals as soon as F- reaches the mitochondrion could explain the increase in GSSG/TGSH ratio due to an augmented amount of peroxides reacting with reduced glutathione and producing higher amounts of oxidized glutathione [35]. The antioxidant enzymes activities were not modified after three pulses of F- and the decreased in GSSG/TGSH could explain the increase in lipid peroxidation after three pulses of F-.

Recently it has been reported that the constant exposure to F- for 72 h is able to induce apoptosis in osteoblasts through increasing oxidative stress. In that work F- was administered to osteoblasts in constant concentration for 72 h [36]. In the experiments described in our paper, osteoblasts are exposed for a few minutes to a high concentration of F-, as it occurs *in vivo*. Although F- levels returned to basal levels 24 h after each pulse of F-, an inhibition of oxygen consumption and respiratory complexes activities and an increase in oxidative stress status were observed.

The results shown in this paper also demonstrate that chronic administration of F- produces a significant decrease in mitochondrial respiratory chain activity. F- treatment significantly inhibited complex IV and partially decreased (30% inhibition) complex I-II and complex I-III activities. As complex I and III are the main sites of superoxide radical synthesis [37], F- could enhance its production by inhibiting the mitochondrial activity at respiratory chain level. F- inhibition at complex IV level could enhance the formation of ubiquinone radical which in turns can react with molecular oxygen increasing superoxide radical production [38]. When the respiratory chain is inhibited (in complex IV in this case), the electron supply reduces the ubiquinone (Q) pool and in the presence of large proton motive force, the electrons are forced back from reduced ubiquinone (QH_2_) into complex I (by electron reverse transport), which has two possible sites of superoxide production: the flavin in the NADH-oxidizing site and the ubiquinone-reducing site [39]. Recently it has been demonstrated that when succinate is used as electron donor, most superoxide is produced at the ubiquinone reduction site [40].

**Figure 5:**
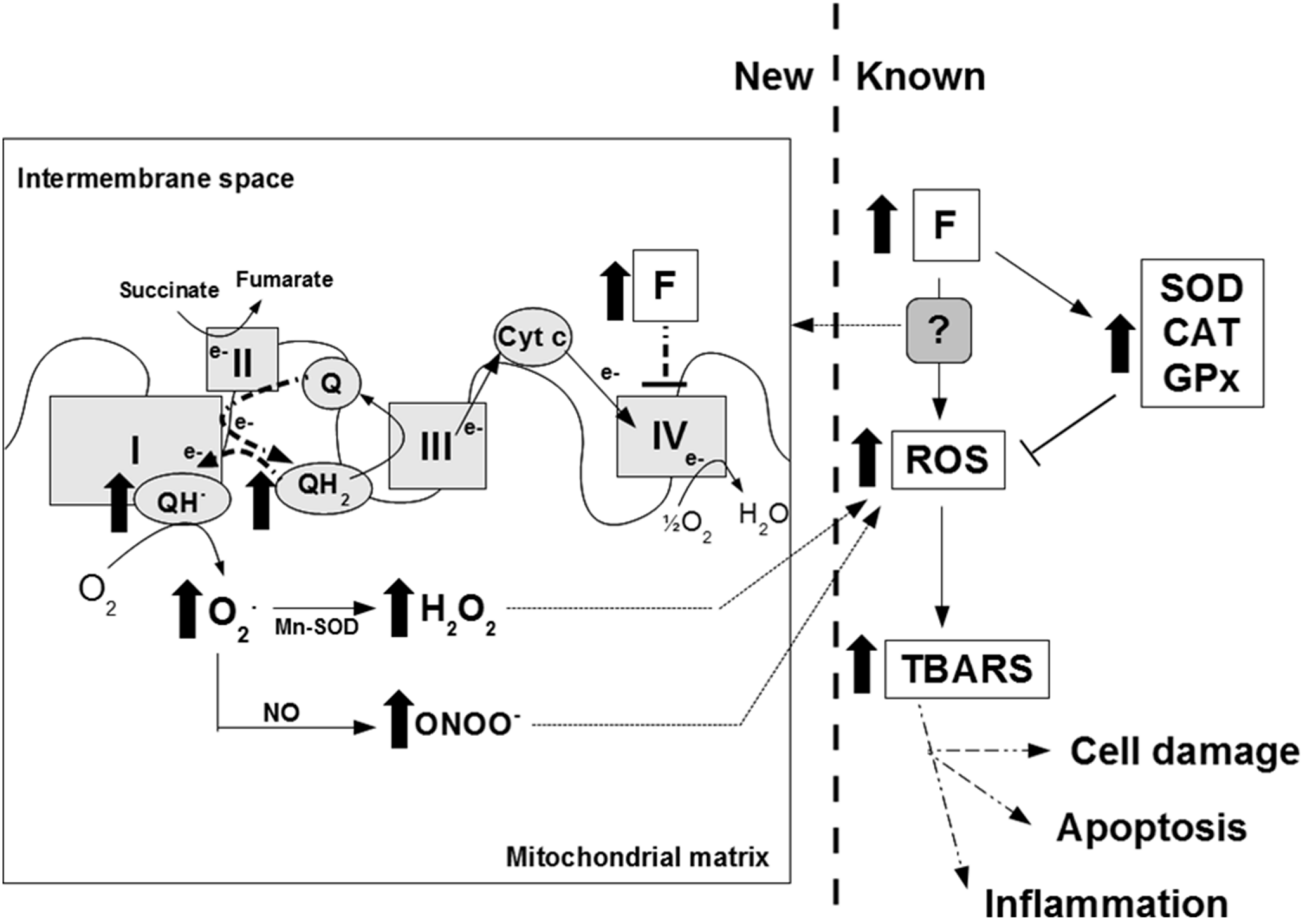
A possible mechanism of fluoride-induced oxygen radicals production. New (left): knowledge generated by this paper. Thick black arrows and thick dashed lines indicate what happens when fluoride arrives at mitochondrial membrane. Known (right): known effects of fluoride on oxidative stress and antioxidant enzymes. F: fluoride; ROS: reactive oxygen species; SOD: superoxide dismutase: CAT: catalase; GPx: glutathione peroxidase; TBARS: thiobarbituric acid reactive substances, I: Complex I-NADH dehydrogenase, II: Complex II-Succinate dehydrogenase, III: Complex III-Cytochrome bc1 complex, IV: Complex IV-Cytochrome c oxidase, cyt c: Cytochrome c, Q: Ubiquinone, QH_2_: Reduced ubiquinone, O_2_^.^: superoxide radical, H_2_O_2_: hydroxide peroxide, NO: Nitric oxide, ONOO^−^: peroxynitrite.

Taking all these results into account, we are able to describe the effect of F- on mitochondrial ROS production and its relationship with oxidative stress and inflammation (Figure 5). After an oral dose, F- may inhibit the respiratory chain, increasing the production of superoxide radical (by the possible mechanism explained before) and thereby of hydroxide peroxide and peroxynitrite [7]. Antioxidant enzymes activities cannot prevent increased free radical formation. Therefore, there is an increase in ROS that finally produce oxidations in membranes and damage the cell macromolecules (as seen by the increase in lipid peroxidation) and may be the cause of the inflammatory foci observed in the bone. It has already been demonstrated that ROS production induced inflammatory gene expression in alveolar macrophages [41], fibroblasts [42] and kidney [43]. Therefore, bone inflammatory foci could be enhanced via ROS-dependent activation of pro-inflammatory genes.

## Supporting Information

Data S1 **Raw data from each figure and table.** In each case, a data table and a legend which explains in detail what the data are, where they come from, the number of repetitions done and the test used for analysis are shown. Regarding the figures, beside each table the corresponding graph is shown.
(XLS)
